# Summaries of harms in systematic reviews are unreliable Paper 1: An introduction to research on harms

**DOI:** 10.1016/j.jclinepi.2021.10.023

**Published:** 2021-11-03

**Authors:** Riaz Qureshi, Evan Mayo-Wilson, Tianjing Li

**Affiliations:** a Department of Epidemiology, Johns Hopkins Bloomberg School of Public Health, Baltimore, MD, USA; b Department of Epidemiology and Biostatistics, Indiana University School of Public Health, Bloomington, ID, USA; c Department of Ophthalmology, University of Colorado Anschutz Medical Campus, Aurora, CO, USA

**Keywords:** Harms, Systematic Reviews, Meta-analysis, Synthesis, Clinical Trials

## Abstract

**Objective:**

Most systematic reviews of interventions focus on potential benefits. Common methods and assumptions that are appropriate for assessing benefits can be inappropriate for harms. This paper provides a primer on researching harms, particularly in systematic reviews.

**Study Design and Setting:**

Commentary describing challenges with assessing harm.

**Results:**

Investigators should be familiar with various terminologies used to describe, classify, and group harms. Published reports of clinical trials include limited information about harms, so systematic reviewers should not depend on these studies and journal articles to reach conclusions about harms. Visualizations might improve communication of multiple dimensions of harms such as severity, relatedness, and timing.

**Conclusion:**

The terminology, classification, detection, collection, and reporting of harms create unique challenges that take time, expertise, and resources to navigate in both primary studies and evidence syntheses. Systematic reviewers might reach incorrect conclusions if they focus on evidence about harms found in published reports of randomized trials of a particular health problem. Systematic reviews could be improved through better identification and reporting of harms in primary studies and through better training and uptake of appropriate methods for synthesizing evidence about harms.

Incomplete knowledge concerning intervention harms threatens public health. Systematic reviews of randomized controlled trials (RCTs) are often thought to provide the most reliable evidence of benefits and harms. Systematic reviews, however, focus largely on potential benefits and typically include trials designed to assess potential benefits. Methods and assumptions that are appropriate for assessing benefits in trials and reviews may be inappropriate for assessing harms. Furthermore, reviews based on publicly available information might be limited because reports often include only a subset of harms, which may be biased.

The sources referenced in this commentary have been collected from regulatory and methods guidance documents, textbooks, non-systematic searching and snowball sampling of journal articles, communication with experts in the field, and discussion among the authors. This paper is the first in a series of three articles (Table 1) dedicated to harms and provides a primer for researching harms and it identifies changes needed to ensure that systematic reviews and clinical practice guidelines consider the trade-offs among all outcomes that matter to patients.

## 1. What are harms?

When patients “weigh the balance of benefits and harms”, they hope that potential positive effects of an intervention will outweigh any negative effects, such that the intervention would be worth taking.[4] “Harms” is an umbrella term for unwanted effects of interventions. This use follows recommendations in the Consolidated Standards of Reporting Trials (CONSORT) Statement Harms extension[5] and the Preferred Reporting Items in Systematic Reviews and Meta-Analyses (PRISMA) Harms extension,[6] which discourage using euphemisms that might obfuscate the importance of these effects. For different interventions and settings, harms might be described as “adverse event(s)”, “adverse effect(s)”, “adverse drug event(s)”, “adverse reaction(s)”, “adverse drug reaction(s)”, “risk(s)”, “safety”, “sequalae”, “toxicity”, “complication(s)”, or “side effect(s).”[7-11]

There are some important distinctions among terms used to describe harms. Box 1 provides some examples. Defining a “harm” also depends on context. That is, an event might be considered a harm in one setting but a beneficial outcome in another; for example, increased weight might be a harm in a trial of an antipsychotic medications or a positive outcome in a trial of an intervention for eating disorders. The same event might also be both a harm and a benefit; for example, a surgical trial might observe both that some people are harmed because they die during surgery and that the surgery is beneficial because it increases overall survival time. Relatedly, not all events are of equal importance to all people; for example, some people might be upset to gain 5% of their body weight while other people might be indifferent or even glad.

## 2. How are harms classified by type and frequency?

Binary or dichotomous harms are often reported as counts or frequencies of events. In regulatory sciences, harms may be collected as events and mapped to standardized terminology before they are analyzed. Examples of standardized terminologies include Common Terminology Criteria for Adverse Events (CTCAE) and Medical Dictionary for Regulatory Activities (MedDRA).[13-15] The Coding Symbols for a Thesaurus of Adverse Reaction Terms (COSTART) preceded MedDRA and was used in trials conducted from 1985 to the late 1990s. MedDRA remains in common use. Both MedDRA and COSTART are hierarchical systems of categorizing harms that (1) establish common vernacular to facilitate analysis and interpretation, and (2) group physiologic events to better describe patterns at higher, aggregated levels.[13,15-19]

**Fig. 1.**
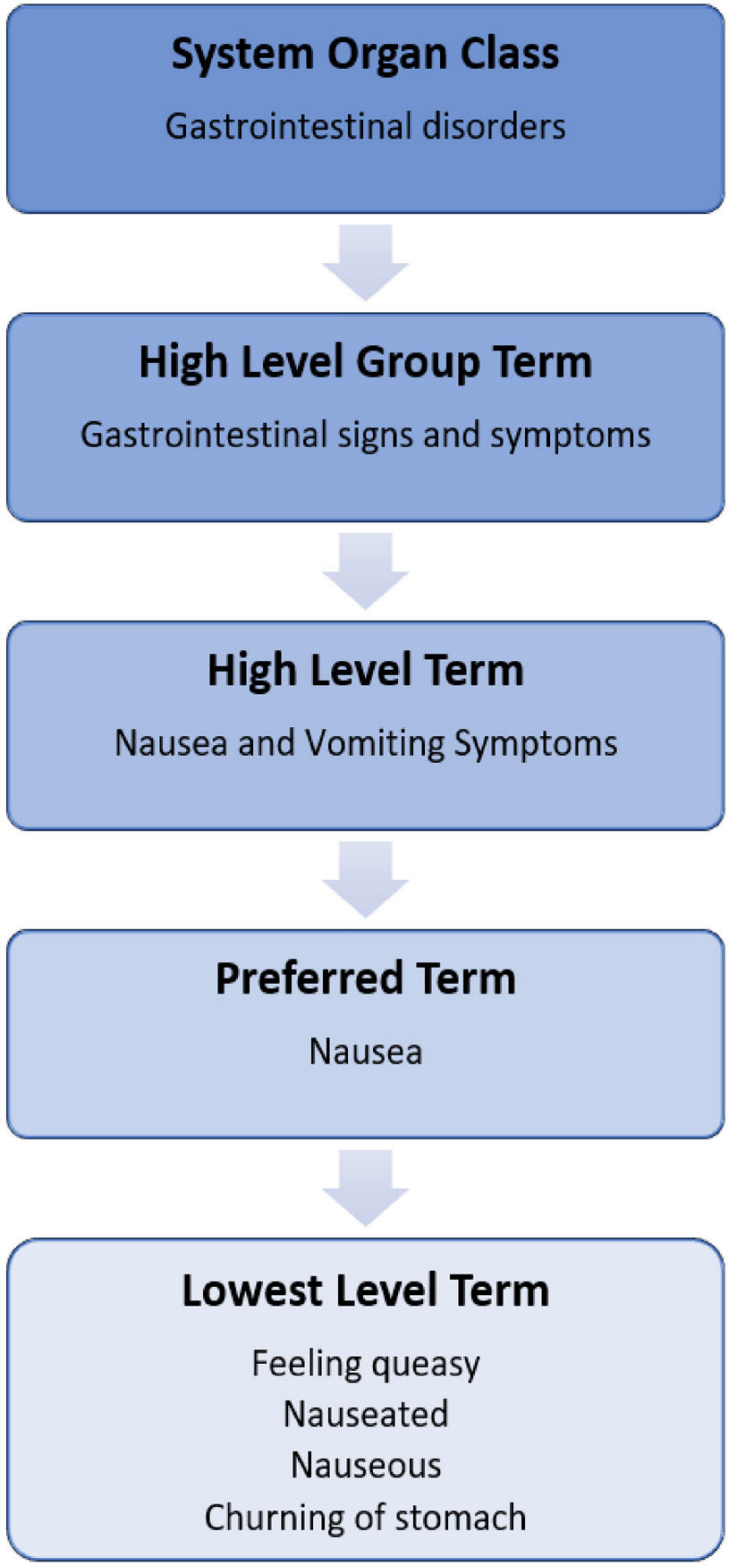
MedDRA hierarchy for nausea.

Patients and clinicians might use multiple terms to describe a single event. For example, an investigator seeking to evaluate whether an intervention makes people drowsy would want to combine similar terms used by patients and clinicians to describe the same feeling.[16-19] MedDRA identifies a number of “lowest level terms” that are considered synonyms for the same “preferred term.” The latter is the standardized code for a specific harm, which is used for the lowest level of analysis.[19] For example, “drowsiness,” “lethargy,” “sedation,” and “somnolence” are ways of referring to the preferred term “somnolence.”[13] Fig. 1 shows an example of the MedDRA path from lowest to higher-level terms for the preferred term “nausea.”[13,19]

Specific events might be related biologically or physiologically, so investigators might want to conduct broader evaluations of harms that share common characteristics.[16-19] For example, assessing harms at higher levels—i.e., above the preferred term—might reveal which body systems are affected (e.g., nervous, genitourinary, cardiac, cardiovascular). Grouping harms in this way has the additional effect of increasing statistical power because, unless individual and specific harms are very common, trials might not observe enough events to precisely estimate harms.[18,20-22] Although different organizations use different cut-offs, Box 2 includes common thresholds for describing the frequency or rarity of harms.[23]

### 2.1. Seriousness and Severity of harms

Related harms might occur at several different levels of severity. For example, allergic reactions can be mild to life-threatening or deadly. The Common Terminology Criteria for Adverse Events (CTCAE) defines five grades of severity (Box 3).[14] “Seriousness” is related to “severity,” but has a specific meaning in regulatory research. That is, a “Serious Adverse Event” is an event that results in (1) death or is life-threatening, (2) hospitalization or prolongation of existing hospitalization, (3) persistent or significant incapacity or substantial disruption of the ability to conduct normal life functions (i.e., disability), or (4) congenital anomaly or birth defect.[12] Legal requirements apply to “serious” harms, which may trigger subsequent actions or reporting requirements to ethics committees and regulatory bodies. While these regulatory and standardized definitions exist, “serious” and “severe” are also commonly used in a non-standardized and informal way, especially outside in studies of unregulated interventions (e.g., clinical psychology, exercise science).

In addition to classifying severity, harms are often classified according to how likely they are to be caused by the intervention. Relatedness is challenging to determine and the approach is not standardized.[24] The challenge of classifying harms data is further exacerbated by the multi-dimensionality of the data as the aspects of timing and repeated events are often ignored when studies analyze harms, although these can play a role in helping to assess relatedness and importance to stakeholders.[25-27] See APPENDIX for more details on relatedness and multi-dimensionality of harms.

## 3. How are harms detected?

Harms can be detected in trials, observational studies, and in clinical care. Different sources of evidence for harms have different strengths and weaknesses with regard to the harms signal that they identify and the certainty of the evidence that they provide for the relationships between the harms and interventions.

### 3.1. Randomized controlled trials

Before a new drug or biologic can be approved for marketing, regulators such as the FDA may require that several studies be conducted, including RCTs. Trials done before marketing approval typically evaluate a drug’s benefits and look for common and clinically important harms.[28] RCTs can provide strong evidence of causality and would be the ideal design to assess harms if designed to do so. In reality, however, RCTs conducted to support regulatory approval often have limitations for harms.[29] For example, harms are rarely chosen as primary outcomes, so RCTs are typically not designed to assess differences in harms between groups. Additionally, RCTs to support regulatory approval often have short duration of follow up with small sample size and observe too few events to detect all but the most common harms (i.e., they are “underpowered”).[20,25,30] Box 4 presents an example to illustrate how many trials are underpowered to detect the true effect size for even common harms.

### 3.2. Observational studies of harms

There are also many different sources of observational data on harms (e.g., cohort studies, case-control studies, case-series, electronic medical records). Compared with RCTs, observational studies face greater challenges stemming from selection bias and information bias. That is, the types of interventions people take for particular health problems might affect the harms that occur for a given intervention in a given population. Despite these limitations, observational studies may be able to achieve larger sample sizes, use less restrictive eligibility criteria (e.g., include people with comorbidities and receiving concomitant interventions), include greater representation of people belonging to minority and underserved populations, and observe longer periods of exposure. These features make observational studies especially useful for detecting unexpected, rare, or long-term harms.[20,29,31-33] Observational studies may be most useful when they are designed to address research questions that RCTs cannot address. Because they can also be very misleading, evidence from RCTs may be more informative when both observational studies and RCTs have addressed the same question. For example, observational studies of hormone replacement therapy for coronary heart disease among postmenopausal women suggested no negative effects of therapy, however large RCTs of the same population, intervention, and outcomes found increased cardiovascular events and a survivorship bias that was missed in the observational studies.[34,35]

Spontaneous reporting systems (i.e., pharmacovigilance) can be valuable for detecting rare or long-term harms of drugs after they have been approved for marketing.[36-38] Examples of these systems include the FDA’s Adverse Event Reporting System (FAERS), Vaccine Adverse Event Reporting System (VAERS), and Sentinel initiative.[39-41] Social media has also been used as a potential source of spontaneously reported harms.[42] FAERS and VAERS are publicly accessible, government-run databases of harms that are reported for any drug and vaccine approved for use in the US, although reports from non-US countries are accepted.[39,40] The majority of reports comprise mandated reporting by manufacturers who must submit any harms reported back to them.[39,40] Reports can also be submitted voluntarily by patients and clinicians. There are several important limitations to this voluntary approach that lead to these surveillance systems being used primarily for hypothesis generation and for identifying potential harms that might warrant further evaluation (Box 5).[36,39,40,43]

In contrast to FAERS and VAERS, which are passive surveillance systems that can be mined for potential drug-harms pairs of interest, Sentinel uses active surveillance of electronic healthcare and administrative claims data. Sentinel is a new program–launched as a pilot in 2008 and fully implemented in 2014–utilizing many databases and surveillance systems. It applies standardized formatting to allow FDA to monitor potential harms of interventions post approval.[41,44,45] Although both types of pharmacovigilance databases have limitations, they are valuable as potential supplemental sources of data for harms in reviews of pharmacologic interventions.[32,46-50]

## 4. How are harms collected?

Unlike benefit outcomes which are typically prespecified in studies, it can be challenging to anticipate every harm that may occur. Harms may be collected systematically (i.e., assessed for all participants using the same scales and instruments at planned times) or non-systematically (i.e., relying on self-reporting by participants).[51-54] See APPENDIX for more details about harms collection.

## 5. How are harms analyzed and reported?

Harms data that have been collected in studies may be insufficient for analyses if they do not include relevant dimensions such as date of onset, duration and severity of the event, and concomitant medications. Additionally, the lack of use of standardized terminology when collecting harms, as described in section 2, can lead to incompleteness and inconsistency in the data which further threatens the validity of harms analyses.[55-60] Interviews with trialists have revealed many reasons for not analyzing and reporting harms, including: an unawareness of the importance of “negative” results, data being considered “uninteresting”, having “too few events worth reporting”, and a perception of limited space in publications”.[55] Indeed, there may even be a purposeful suppression of the evidence of harm; an anonymous trialist interviewed by Smyth and colleagues in 2010 said:

“When we looked at that data, it actually showed an increase in harm amongst those who got the active treatment, and we ditched it because we weren’t expecting it and we were concerned that the presentation of these data would have an impact on people’s understanding of the study findings.” [85]

Improving the reporting of harms in trials is an ongoing challenge that remains unsolved, despite the recommendations of the CONSORT-Harms extension being 16 years old.[61]

### 5.1. Use of selection criteria to report harms

Most reports about interventions–from journal articles to FDA prescribing information–do not enumerate all harms that are recorded in trials and observational studies.[62] Instead, authors “selection criteria,” which are rules to determine which harms will be presented in a given report.[62] Authors might choose a subset of all harms based on frequency or based on perceived importance to patients and clinicians.[62] Selection criteria might be used to limit the number of harms to be reported because of space constraints or because readers would find it difficult to interpret hundreds of effect estimates.[5,55,62] Because selection criteria restrict what is reported to a small fraction of what was found in a study, they can be used to purposely supress certain findings. Biased reporting could be mitigated by pre-specifying which harms will be reported, however, investigators do not always know which important harms will occur before the data are collected. Moreover, selection criteria are variable between trial reports and within multiple data sources about the same trial (e.g., trial registration, drug label, publication).[62] Thresholds for reporting create a specific challenge for synthesis because reported harms are typically those that are “very common” (Box 2); because studies with null or small effects are systematically missing from the literature, meta-analyses will tend to be biased.

**Fig. 2.**
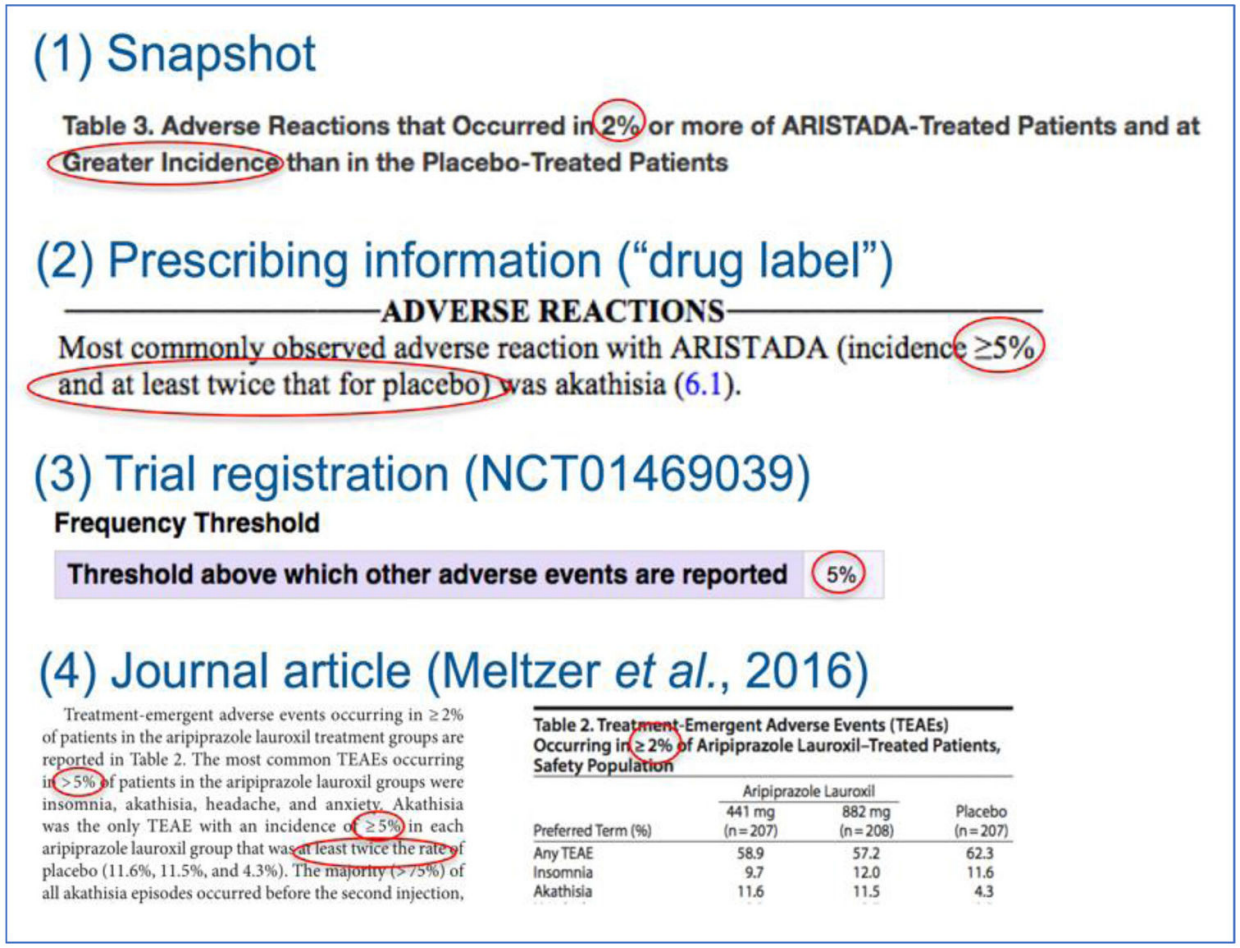
Inconsistent selection criteria applied across different sources for the same trial.

Fig. 2 presents several examples of selection criteria from different sources (i.e., drug label, trial registration, and journal article) with multiple thresholds for a single trial of Aristada (aripiprazole lauroxil).[63-65] These different rules in reports that rely on data from the same trials mean that different harms are presented to evidence users depending on where they look.[62,66,67]

The extent to which harms are underreported using various selection criteria can be examined by comparing different public sources for trials with the individual participant data: the more restrictive the selection criteria, the less of the overall picture consumers, clinicians, and systematic reviews have when they do not have access to the full, unpublished, data.[62,68,69] Individual participant data (IPD) for trials can sometimes be obtained through data sharing sites such as Vivli or YODA (Yale Open Data Access), government repositories such as European Medicine Agency Clinical Data or Health Canada Clinical Information on Drugs and Health Products, or from the clinical study reports supplied by the pharmaceutical manufacturers.[70-73] Clinical study reports are the most comprehensive study documents often created by a pharmaceutical manufacturer for submission to a regulator.[74,75] They detail the design, methods, analyses, and results of a study.[74-77] Appendices of CSRs sometimes contain tables of individual patient data, also called “patient data listings”, and study protocols.[74-77]

## 6. How do systematic reviews assess harms?

The limitations of harms assessment, analysis, and reporting in individual studies affect evidence syntheses and clinical practice guidelines. The evidence provided by a systematic review can only be as good as the evidence in the included studies. If the limitations of available data are not considered in the review process, then a review might reach incorrect conclusions concerning harms and the trade-offs between benefits and harms. Although the current paradigm for conducting systematic reviews of interventions recommends that harms be assessed so that there can be a balanced discussion of potential benefits and harms, most reviews are not designed to assess harms rigorously[6,11,78-80] and might reach conclusions that are misleading or wrong. Early assessments of systematic review methods and reporting of harms revealed limitations in the approaches taken by reviewers to assessing harms at each stage of the review process, including: restrictions on the sources that are searched for evidence and the types of studies included, limitations in the analyses of harms, and poor reporting of methods used to assess harms (Box 6).[58,78-86]

## 7. What are the recommendations for systematic reviewers in assessing harms?

Based on the early assessments of how systematic reviews synthesized harms, guidance has been developed for searching for evidence, performing analysis, and reporting results. A 2008 report by the Agency for Health-care Research and Quality[32] was followed by the *Institute of Medicine’s Finding What Works in Health Care: Standards for Systematic Reviews* and the first edition of the *Cochrane Handbook for Systematic Reviews of Interventions*, both published in 2011.[87,88] These provided guidance for incorporating harms in the review process. Later, the 2015 PRISMA-Harms extension and a 2018 FDA Guidance for Industry document provided more specific guidance on reporting harms in reviews and for conducting meta-analyses of harms.[6,89] The 2nd edition of the Cochrane Handbook, published in 2020, includes more detailed recommendations about including harms in reviews.[11,90] Although these sources focus on different aspects of the review process, there are several common themes that are consistent in their recommendations (Box 7).

Implementing these recommendations requires a substantial investment, both in time and resources. For example, the time required to obtain and make use of unpublished data can be prohibitive for reviewers because harmonizing IPD is labor intensive and because important details may be buried in thousands of pages of reports.[75] Additionally, the expertise necessary to implement some recommendations, such as appropriately incorporating observational data, may be beyond what some review teams possess.

## 8. Conclusions

Assessing harms is a demanding process that requires much work and careful thought, above and beyond that required to synthesize evidence of efficacy or effectiveness. The terminology, classification, detection, collection, and reporting of harms create unique challenges that take time, expertise, and resources to yield valid summaries of harms. Investigators of primary studies should be mindful of these issues as their implementation can greatly impact subsequent evidence synthesis. To avoid tokenism and incorrect conclusions for harms, systematic reviewers should carefully consider how to handle each of these aspects if they plan to draw conclusions about trade-offs between benefits and harms. More systematic reviews are needed that focus exclusively on harms, include appropriate study designs, populations, and data sources, and that apply valid methods for synthesis.

## Supplementary Material

Appendix

## Figures and Tables

**Table 1.** Synthesis of harms: Challenges and opportunities

Series paper	Description
Qureshi R, Mayo-Wilson E, Li T. Summaries of harms in systematic reviews are unreliable Paper 1: An introduction to research on harms.[1]	This paper describes key aspects of assessing harms. First, it identifies the various ways that harms are classified, including the terminology used to describe grouping of harms and different data dimensions such as rarity, severity, relatedness, and timing. Second, it explores different methods for assessing harms in clinical research. Third, it describes the challenges associated with analyzing, reporting, and synthesizing harms.
Qureshi R, Mayo-Wilson E, Rittiphairoj T, McAdams-DeMarco M, Guallar E, Li T. Summaries of harms in systematic reviews are unreliable Paper 2: Methods used to assess harms are neglected in systematic reviews of gabapentin.[2]	Part 1 of an overview of gabapentin systematic reviews that serves as a methodologic study for how harms are synthesized. This paper examines the methods used in systematic reviews and meta-analyses to assess harms across all stages of the review process and compared these with recommendations for assessing harms in reviews. We found review methods are focused on addressing questions of benefit and the tokenistic inclusion of harms in reviews means that the methods may not meet the needs to produce a valid synthesis of harms.
Qureshi R, Mayo-Wilson E, Rittiphairoj T, McAdams-DeMarco M, Guallar E, Li T. Summaries of harms in systematic reviews are unreliable Paper 3: Given the same data sources, systematic reviews of gabapentin have different results for harms.[3]	Part 2 of an overview of gabapentin systematic reviews that serves as a methodologic study for how harms are synthesized. This paper evaluates the results for harms across these reviews, with a particular interest in the consistency of results given similar supporting data. We found significant challenges in the selection of harms to assess and report, largely driven by reviewer preferences as opposed to standardized approaches, leading to different summaries of harms across reviews.

## What is new?

- Valid and reliable syntheses of evidence about harms requires different types of data, and different methods for synthesis, compared with evidence about benefits.
- The terminology, classification, detection, collection, and reporting of harms create unique challenges which take time, training and expertise, and resources to navigate.
- Systematic reviewers should carefully consider how to handle each of these aspects if they plan to synthesize harms or to draw conclusions about trade-offs between benefits and harms.
- In systematic reviews that focus on potential intervention benefits, methods to identify and synthesize evidence about harms are often tokenistic.
- Many interventions (e.g., drugs) are used for multiple indications (i.e., health problems), and harms might be related to interventions rather than indications. Consequently, systematic reviews focused exclusively on harms might be the most comprehensive and valid approach to synthesize evidence across indications using multiple study designs and data sources.

## Box 1 Different definitions within the umbrella of “harms”

### Adverse event

United States (US) Food and Drug Administration (FDA) defines “adverse event” as: “Any untoward medical occurrence associated with the use of a drug in humans, whether or not considered drug related.”[12]

Similar definitions are shared by other agencies around the world such as the International Conference on Harmonization (ICH), European Medical Association (EMA), and World Health Organization (WHO).

### Side effect

On the other hand, “side effects” are defined very differently by the WHO as:

“Any unintended effect of a pharmaceutical product occurring at doses normally used in humans which is related to the pharmacological properties of the medicine. Such effect may be either positive or negative. Such effects may be well-known and even expected and may require little or no change in patient management.”[7]

Whereas “adverse events” do not have to be causally related to interventions and are always negative, “adverse effects” are also negative but are related to an intervention, and “side effects” are related to the intervention and can be either negative or positive.[10,11] For example, a potentially desirable side effect was found for sildenafil (Viagra), which was originally intended as a blood pressure medication and had a strong unintended “side effect” that led to its use for erectile disfunction.

## Box 2 Council for International Organizations of Medical Sciences thresholds of occurrence

Very common ≥ 1/10

Common (frequent) < 1/10 AND ≥ 1/100

Uncommon (infrequent) < 1/100 AND ≥ 1/1000

Rare < 1/1000 AND ≥ 1/10000

Very rare < 1/10000

## Box 3 Grades to classify severity of harms

Grade 1 – “Mild”: Asymptomatic or minor symptoms; clinical or diagnostic observations only; no intervention needed

Grade 2 – “Moderate”: Minimal, local, or non-invasive intervention indicated

Grade 3 – “Severe”: Medically significant but not immediately life-threatening; hospitalization or prolongation of hospitalization indicated; disabling

Grade 4 – Life-threatening consequences (i.e., immediate risk of death); urgent intervention indicated

Grade 5 – Death related to adverse event

## Box 4 Example of inadequate power for common harms in Phase 3 trials

Assume that a randomized trial (e.g., conducted to seek marketing approval) has a sample size of 300 patients, a 1:1 allocation, and treatment duration of 6 months.

Assume a “common” harm has a true annual incidence of 1/50 among those receiving Intervention. The harm is three times as common in the Intervention group compared with the Control group (i.e., the true Risk Ratio = 3).

In this hypothetical trial, on average, we could expect 1.5 events in the Intervention group and 0.5 events in the Control group. It would take a trial twice the size before we could expect to see a single “common” event in the Control group.

In a trial such as this, in which 0 to 2 events might be expected in either group, the observed Risk Ratio will often be 1, infinitely positive, or infinitely negative.

Following conventions for estimating statical power for benefit outcomes, a trial approximately eight times this size (i.e., over 2300 patients) would be needed to precisely estimate a “common” harm such as this.

## Box 5 Limitations of assessing harms using spontaneous reporting systems

- Information biases (such as recall biases and sub-optimal reporting quality);
- Confounding by indication: Harms might be associated a drug because they are causally associated with the condition for which the drug was prescribed;
- Polypharmacy: Harms might be caused by concomitant medications that are associated with the drug under investigation;
- Issues with data accuracy and completeness in reports (e.g., missing demographics or timing);
- The possibility of duplicate reports (i.e., reports of the same event submitted by both a patient and their doctor);
- Information in the reports has not been verified (i.e., submission of a report does not mean the information has been medically confirmed); and
- Common measures of effect such as rate and incidence cannot be determined because there is no denominator for the total number of people or person time exposed to a drug.

## Box 6 Common limitations in harm assessment in systematic reviews

- Reviews designed to assess the potential benefits of an intervention for a particular health problem (e.g., sertraline for adolescents with depression) tend to exclude evidence about potential harms from relevant studies, including preclinical studies (e.g., studies in non-human animals), clinical studies of related interventions (e.g., studies of similar antidepressants), studies of the same intervention for different health problems (e.g., sertraline for anxiety), and studies in related populations (e.g., adults with depression).
- Systematic reviewers limit evidence searches to bibliographic databases, and searches are poorly constructed and unlikely to return evidence for harms.
- Unpublished data are rarely used in reviews, and reviewers often restrict sources of evidence to the primary publications of RCTs.
- With limited research on the comparability and validity of models and assumptions for meta-analysis of rare events, reviewers commonly use default methods of meta-analysis.
- The reporting of how reviewers assessed harms is generally poor and inconsistent.

## Box 7 Recommendations for methods to assess harms in systematic reviews

- Assess multiple measures for harm including multiple specific harms (i.e., preferred terms), general harms (e.g., “risk of any harm”), and proxies for harm (e.g., “drop out due to harm”)
- Focus on specific harms (as opposed to general assessments of harm)
- Search multiple sources of evidence (e.g., bibliographic databases, grey literature, etc.) to identify information about harms.
- Do not restrict the search for evidence to RCTs: look for harms data in observational studies, case reports, and adverse event reporting systems and assess the quality of the data appropriately.
- Utilize any unpublished data that can be obtained for included studies (e.g., Clinical Study Reports).
- Consider absolute measures (e.g., risk difference) over relative measures (e.g., risk ratio and odds ratio) when conducting a meta-analysis because they may give a better indication of public health impact, however they have wider confidence intervals and may have lower statistical power for rare events.
- For rare harms, including those with zero events in one or more arms, avoid inverse-variance and Dersimonian and Laird methods when selecting a model for meta-analysis and give preference to Bayesian or select frequentist models (e.g., Peto one-step odds ratio or Mantel-Haenszel odds ratio without zero-cell corrections).
- Report methods for assessing harms in systematic reviews, including details of how the primary studies assessed harms.

## References

[1] Qureshi R , Mayo-Wilson E , Li T . Summaries of harms in systematic reviews are unreliable Paper 1: An introduction to research on harms. J Clin Epidemiol 2021 (IN PRESS). 10.1016/j.jclinepi.2021.10.023 PMC9126149 34742788

[2] Qureshi R, Mayo-Wilson E, Rittiphairoj T, McAdams-DeMarco M, Guallar E, Li T. Summaries of harms in systematic reviews are unreliable Paper 2: Methods used to assess harms are neglected in systematic reviews of gabapentin. J Clin Epidemiol 2021 *(IN PRESS)*. 10.1016/j.jclinepi.2021.10.024 PMC9875742 34742789

[3] Qureshi R, Mayo-Wilson E, Rittiphairoj T, McAdams-DeMarco M, Guallar E, Li T. Summaries of harms in systematic reviews are unreliable Paper 3: Given the same data sources, systematic reviews of gabapentin have different results for harms. J Clin Epidemiol 2021 *(IN PRESS)*. 10.1016/j.jclinepi.2021.10.025 PMC9875741 34742790

[4] Bougioukas KI, Liakos A, Tsapas A, Ntzani E, Haidich AB. Preferred reporting items for overviews of systematic reviews including harms checklist: a pilot tool to be used for balanced reporting of benefits and harms. J Clin Epidemiol 2018;93:9–24. doi:10.1016/j.jclinepi.2017.10.002. 29037888

[5] Ioannidis JP, Evans SJ, Gøtzsche PC, O’Neill RT, Altman DG, Schulz K, Improving patient care better reporting of harms in randomized trials : An extension of the CONSORT statement. Ann Intern Med 2004;141(10):781–8. doi:10.7326/0003-4819-141-10-200411160-00009. 15545678

[6] Zorzela L, Loke YK, Ioannidis JPA, Golder S, Santaguida PL, Altman DG, PRISMA Harms: improving harms reporting in systematic reviews. Br Med J 2016;352(157):1–17. doi:10.1136/bmj.i157. 26830668

[7] Management Sciences for Health and World Health Organization. Drug and Therapeutics Committee Training Course: Assessing and Managing Medicine Safety. Vol Submitted.; 2007. Accessed May 5, 2021. https://www.who.int/medicines/technical_briefing/tbs/04-PG_Dug-Safety_final-08.pdf?ua1#:~:text= Adverse drug reaction (ADR)—,the modification of physiological function.”

[8] Lindquist M The need for definitions in pharmacovigilance. Drug Saf 2007;30(10):825–30. doi:10.2165/00002018-200730100-00001. 17867720

[9] European Medicines Agency. Guideline on good pharmacovigilance practices (GVP) Annex I - Definitions (Rev 4). 2017;(October):1–47. doi:EMA/876333/2011 Rev. 1*

[10] Aronson JK. Distinguishing hazards and harms, adverse drug effects and adverse drug reactions: Implications for drug development, clinical trials, pharmacovigilance, biomarkers, and monitoring. Drug Saf 2013;36(3):147–53. doi:10.1007/s40264-013-0019-9. 23417506

[11] Peryer G, Golder S, Junqueira D, Vohra S, Kong Loke Y. Chapter 19: Adverse effects. Cochrane Handbook for Systematic Reviews of Interventions. Higgins J, Thomas J, Chandler J, Cumpston M, Li T, Page MVAW, editors; 2019. Version 6. Cochrane https://training.cochrane.org/handbook/version-6/chapter-19-draftv2.

[12] US department of Health & Human Services. CFR Title 21, Section 312.32. IND Safety Reporting. 2010. https://www.accessdata.fda.gov/scripts/cdrh/cfdocs/cfcfr/CFRSearch.cfm?fr=312.32

[13] International Conference on Harmonization. MedDRA Medical Dictionary for Regulatory Activities. ICH Official Web Site. Published 2021. Accessed May 6, 2021. https://www.ich.org/page/meddra

[14] National Cancer Institute Common Terminology Criteria for Adverse Events; 2020. doi:1032388/erjxiq.

[15] U.S Food and Drug Administration (FDA) National Adverse Drug Reaction Directory: “COSTART” (Coding Symbols for Thesaurus of Adverse Reaction Terms). US Department of Health, Education, and Welfare; 1970.

[16] Andrews JC, Bogliatto F, Lawson HW, Bornstein J. Speaking the same language: Using standardized terminology. J Low Genit Tract Dis 2016;20(1):8–10. doi:10.1097/LGT.0000000000000157. 26579837

[17] Fizames C How to improve the medical quality of the coding reports based on who-art and costart use. Ther Innov Regul Sci 1997;31(1):85–92. doi:10.1177/009286159703100112.

[18] Tremmel LT, Scarpone L. Using MedDRA for adverse events in cancer trials: Experience, caveats, and advice. Ther Innov Regul Sci 2001;35(3):845–52. doi:10.1177/009286150103500322.

[19] International Conference on Harmonization. MedDRA Hierarchy - How to use. Published 2016. Accessed May 6, 2021. http://www.meddra.org/how-to-use/basics/hierarchy

[20] Tsang R, Colley L, Lynd LD. Inadequate statistical power to detect clinically significant differences in adverse event rates in randomized controlled trials. J Clin Epidemiol 2009;62:609–16. doi:10.1016/j.jclinepi.2008.08.005. 19013761

[21] Ernst E, Pittler MH. Assessment of therapeutic safety in systematic reviews: Literature review. Br Med J 2001;323(7312):546. doi:10.1136/bmj.323.7312.546. 11546700 PMC48159

[22] Bennetts M, Whalen E, Ahadieh S, Cappelleri JC. An appraisal of meta-analysis guidelines: How do they relate to safety outcomes? Res Synth Methods 2017;8(1):64–78. doi:10.1002/jrsm.1219. 27612447

[R23] Council for International Organizations of Medical SciencesGuidelines for Preparing Core Clinical-Safety Information on Drugs: Report of CIOMS Working Groups III and V – Including New Proposals for Investigator’s Brochures; 1999.

[24] Le-Rademacher J, Hillman SL, Meyers J, Loprinzi CL, Limburg PJ, Mandrekar SJ. Statistical controversies in clinical research: Value of adverse events relatedness to study treatment: Analyses of data from randomized double-blind placebo-controlled clinical trials. Ann Oncol 2017;28(6):1183–90. doi:10.1093/annonc/mdx043. 28184420

[25] Ma H, Ke C, Jiang Q, Snapinn S. Statistical considerations on the evaluation of imbalances of adverse events in randomized clinical trials. Ther Innov Regul Sci 2015;49(6):957–65. doi:10.1177/2168479015587363. 30222385

[26] Duke JD, Li X, Grannis SJ. Data visualization speeds review of potential adverse drug events in patients on multiple medications. J Biomed Inform 2010;43(2):326–31. doi:10.1016/j.jbi.2009.12.001. 19995616 PMC2838979

[27] Tarn DM, Wenger A, Good JS, Hoffing M, Scherger JE, Wenger NS. Do physicians communicate the adverse effects of medications that older patients want to hear? Drugs Ther Perspect 2015;31(2):68–76. doi: 10.1007/s40267-014-0176-7. 25750513 PMC4347985

[28] Food and Drug Administration. The FDA’s drug review process: ensuring drugs are safe and effective. FDA Information for Consumers. Published 2018. Accessed December 5, 2018. https://www.fda.gov/Drugs/ResourcesForYou/Consumers/ucm143534.htm

[29] Hammad TA, Pinheiro SP, Neyarapally GA. Secondary use of randomized controlled trials to evaluate drug safety: A review of methodological considerations. Clin Trials 2011;8(5):559–70. doi:10.1177/1740774511419165. 21878445

[30] Zink RC, Marchenko O, Sanchez-Kam M, Ma H, Jiang Q. Sources of safety data and statistical strategies for design and analysis: Clinical trials. Ther Innov Regul Sci 2018;52(2):141–58. doi:10.1177/2168479017738980. 29714519

[31] Papanikolaou PN, Christidi GD, Ioannidis JP. Comparison of evidence on harms of medical interventions in randomized and non-randomized studies. J Can Med Assoc 2006;174(5):635–41. doi:10.1503/cmaj.050873. PMC1389826 16505459

[32] Chou R, Aronson N, Atkins D, Ismaila A, Santaguida P, Smith D, Assessing Harms When Comparing Medical Interventions: Methods Reference Guide for Effectiveness and Comparative Effectiveness Reviews.; 2008. doi:10.1016/j.jclinepi.2008.06.007 21433406

[33] Vandenbroucke JP. When are observational studies as credible as randomised trials? Lancet 2004;363:1728–31. doi:10.1016/S0140-6736(04)16261-2. 15158638

[34] Manson J, Chlebowski R, Stefanick M, Aragaki A, Rossouw J, Prentice R, Women’s Health Initiative hormone therapy trials: Update and overview of health outcomes during the intervention and post-stopping phases. JAMA 2013;310(13):1353–68. doi:10.1001/jama.2013.278040. 24084921 PMC3963523

[35] Vandenbroucke JP. The HRT controversy: Observational studies and RCTs fall in line. Lancet 2009;373(11):1233–5. doi:10.1016/S0140-6736(09)60708-X. 19362661

[36] Toki T, Ono S. Spontaneous reporting on adverse events by consumers in the United States: an analysis of the food and drug administration adverse event reporting system database. Drugs - Real World Outcomes 2018;5(2):117–28. doi:10.1007/s40801-018-0134-0. 29725886 PMC5984610

[37] Duggirala HJ, Tonning JM, Smith E, Bright RA, Baker JD, Ball R, Data Mining at FDA 2015:1–24. (Cvm) http://www.fda.gov/downloads/scienceresearch/dataminingatfda/ucm443675.pdf.

[38] Pandey A, Kreimeyer K, Foster M, Botsis T, Dang O, Ly T, Adverse event extraction from structured product labels using the Event-based Text-mining of Health Electronic Records (ETHER)system. Health Informatics J 2018;00(0). doi:10.1177/1460458217749883. 29359620

[39] Food and Drug Administration. Questions and answers on FDA’s Adverse Event Reporting System (FAERS). FDA Adverse Events Reporting System (FAERS). Published 2018. Accessed December 5, 2018. https://www.fda.gov/Drugs/ResourcesForYou/Consumers/ucm143534.htm

[40] US department of Health & Human Services. VAERS Data Use Guide.; 2017. https://vaers.hhs.gov/docs/VAERSDataUseGuide_October2017.pdf

[41] U.S Food and Drug Administration (FDA). FDA’s Sentinel Initiative. FDA’s Sentinel Initiative. Accessed May 6, 2021. www.fda.gov/safety/fdas-sentinel-initiative

[42] Lee J-Y, Lee Y-S, Kim D, Lee H, Yang B, Kim M . The use of social media in detecting drug safety–related new black box warnings, labeling changes, or withdrawals: scoping review. J Med Internet Res Public Heal Surveill 2021;7(6):e30137. 10.2196/30137 PMC8277336 34185021

[43] Geier DA, Geier MR. A review of the vaccine adverse event reporting system database. Expert Opin Pharmacother 2004;5(3):691–8. doi:10.1517/14656566.5.3.691. 15013937

[44] Ball R, Robb M, Anderson SA, Dal Pan G. The FDAs sentinel initiative A comprehensive approach to medical product surveillance. Clin Pharmacol Ther 2016;99(3):265–8. doi:10.1002/cpt.320. 26667601

[45] Platt R, Brown J, Robb M, McClellan M, Ball R, Nguyen M, The FDA Sentinel Initiative – An evolving national resource. N Engl J Med 2018;379(22):2091–3. doi:10.1056/NEJMp1809643, 30485777

[46] Grothen A, Rivera D. Reviewing adverse drug event reporting between randomized clinical trial data and real world post market data for Sorafenib and Sunitinib. Value Heal 2018;21:S14. doi:10.1016/j.jval.2018.04.081.

[47] Smith K, Golder S, Sarker A, Loke Y, O’Connor K, Gonzalez-Hernandez G. Methods to compare adverse events in twitter to FAERS, drug information databases, and systematic reviews: Proof of concept with adalimumab. Drug Saf 2018;41(12):1397–410. doi:10.1007/s40264-018-0707-6. 30167992 PMC6223697

[48] Anyanwagu U, Mamza J, Gordon J, Donnelly R, Idris I. Premixed vs basal-bolus insulin regimen in Type 2 diabetes: Comparison of clinical outcomes from randomized controlled trials and real-world data. Diabet Med 2017;34(12):1728–36. doi:10.1111/dme.13518. 28945928

[49] Kibbelaar RE, Oortgiesen BE, van der Wal-Oost AM, Boslooper K, Coebergh JW, Veeger NJGM, Bridging the gap between the randomised clinical trial world and the real world by combination of population-based registry and electronic health record data: A case study in haemato-oncology. Eur J Cancer 2017;86:178–85. doi:10.1016/j.ejca.2017.09.007. 28992561

[50] Berger ML, Sox H, Willke RJ, Brixner DL, Eichler HG, Goettsch W, Madigan D, Good practices for real-world data studies of treatment and/or comparative effectiveness: Recommendations from the joint ISPOR-ISPE Special Task Force on Real-World Evidence in Health Care Decision Making. Value Heal 2017;20(8):1003–8. doi:10.1016/j.jval.2017.08.3019. 28964430

[51] Mayo-Wilson E, Fusco N, Li T, Hong H, Canner JK, Dickersin K. Multiple outcomes and analyses in clinical trials create challenges for interpretation and research synthesis. J Clin Epidemiol 2017;86:39–50. doi:10.1016/j.jclinepi.2017.05.007. 28529187

[52] Mayo-Wilson E, Li T, Fusco N, Bertizzolo L, Canner JK, Cowley T, Cherry-picking by trialists and meta-analysts can drive conclusions about intervention efficacy. J Clin Epidemiol 2017;91:95–110. doi:10.1016/j.jclinepi.2017.07.014. 28842290

[53] Zarin DA, Tse T, Williams RJ, Carr S. Trial reporting in ClinicalTrials.gov – The final rule. N Engl J Med 2016;375(20):1998–2004. doi:10.1056/NJMsr1611785. 27635471 PMC5225905

[54] US department of Health & Human Services Clinical trials registration and results information submission: final rule. Fed Regist 2016;81(183):64981–5157. doi:10.17226/10028. 27658315

[55] Smyth R, Kirkham JJ, Jacoby A, Altman DG, Gamble C, Williamson P. Frequency and reasons for outcome reporting bias in clinical trials: Interviews with trialists. Br Med J 2010;341:c7153. doi:10.1136/bmj.c7153. PMC3016816 21212122

[56] Hodkinson A, Kirkham JJ, Tudur-Smith C, Gamble C. Reporting of harms data in RCTs: A systematic review of empirical assessments against the CONSORT harms extension. Br Med J Open 2013;3:e003436. doi:10.1136/bmjopen-2013-003436. PMC3787508 24078752

[57] Hernandez A, Walker E, Ioannidis JPA, Kattan MW. Challenges in meta-analysis of randomized clinical trials for rare harmful cardiovascular events: The case of rosiglitazone. Am Heart J 2008;156(1):23–30. doi:10.1016/j.ahj.2008.03.002. 18585493

[58] Saini P, Loke YK, Gamble C, Altman DG, Williamson PR, Kirkham JJ. Selective reporting bias of harm outcomes within studies: Findings from a cohort of systematic reviews. Br Med J 2014;349:g6501. doi:10.1136/bmj.g6501. 25416499 PMC4240443

[59] Kirkham JJ, Dwan KM, Altman DG, Gamble C, Dodd S, Smyth R, The impact of outcome reporting bias in randomised controlled trials on a cohort of systematic reviews. Br Med J 2010;340:c365. doi:10.1136/bmj.c365. 20156912

[60] Kirkham JJ, Riley RD, Williamson PR. A multivariate meta-analysis approach for reducing the impact of outcome reporting bias in systematic reviews. Stat Med 2012;31:2179–95. doi:10.1002/sim.5356. 22532016

[61] Junqueira DR, Phillips R, Zorzela L, Golder S, Loke Y, Moher D, Time to improve the reporting of harms in randomized controlled trials. J Clin Epidemiol 2021;136:216–20. doi:10.1016/j.jclinepi.2021.04.020. 33984494

[62] Mayo-Wilson E, Fusco N, Hong H, Li T, Canner JK, Dickersin K. Opportunities for selective reporting of harms in randomized clinical trials: Selection criteria for non-systematic adverse events. Trials 2019;20(1):553. doi:10.1186/s13063-019-3581-3, 31488200 PMC6728982

[63] NCT01469039. A study to evaluate the efficacy and safety of ALKS 9072 (Also known as Aripiprazole lauroxil, ALKS 9070, or ARIS-TADA) in subjects with Schizophrenia. ClinicalTrials.gov. Published 2016. https://clinicaltrials.gov/ct2/show/results/NCT01469039

[64] Food and Drug Administration. Label: Aripiprazole Lauroxil (ARIS-TADA).; 2015. https://www.accessdata.fda.gov/drugsatfda_docs/label/2015/207533s000lbl.pdf

[65] Meltzer HY, Risinger R, Nasrallah HA, Du Y, Zummo J, Corey L, A randomized, double-blind, placebo-controlled trial of aripiprazole lauroxil in acute exacerbation of schizophrenia. J Clin Psychiatry 2015;76(8):1085–90. doi:10.4088/JCP.14m09741. 26114240

[66] Mayo-Wilson E, Li T, Fusco N, Dickersin K. Practical guidance for using multiple data sources in systematic reviews and meta-analyses (with examples from the MUDS study). Res Synth Methods 2017:1–11 Published online 2017. doi:10.1002/jrsm.1277, PMC5888128 29057573

[67] Li T, Mayo-Wilson E, Fusco N, Hong H, Dickersin K. Caveat emptor: the combined effects of multiplicity and selective reporting. Trials 2018;19(1):4–9. doi:10.1186/s13063-018-2888-9. 30223876 PMC6142307

[68] Mayo-Wilson E, Fusco N, Li T, Hong H, Canner JK, Dickersin K. Harms are assessed inconsistently and reported inadequately Part 1: Systematic adverse events. J Clin Epidemiol 2019;113:20–7. doi:10.1016/j.jclinepi.2019.04.022. 31055175

[69] Mayo-Wilson E, Fusco N, Li T, Hong H, Canner JK, Dickersin K. Harms are assessed inconsistently and reported inadequately Part 2: Non-systematic adverse events. J Clin Epidemiol 2019;113:11–19. doi:10.1016/j.jclinepi.2019.04.020. 31055176

[70] Health Canada. Clinical information on drugs and health products. Published 2021. Accessed May 15, 2021. https://clinical-information.canada.ca/search/ci-rc

[71] European Medicines Agency. EMA Clinical Data. Published 2021. Accessed May 15, 2021. https://clinicaldata.ema.europa.eu/web/cdp

[72] Yale Open Data Access. The YODA Project. Published 2021. Accessed May 15, 2021. https://yoda.yale.edu

[73] Vivli Center for Global Research Data. Vivli.org. Published 2021. Accessed May 15, 2021. https://vivli.org

[74] Wieseler B, Wolfram N, McGauran N, Kerekes MF, Vervölgyi V, Kohlepp P, Completeness of reporting of patient-relevant clinical trial outcomes: Comparison of unpublished clinical study reports with publicly available data. PLoS Med 2013;10(10):e1001526. doi:10.1371/journal.pmed.1001526. 24115912 PMC3793003

[75] Doshi P, Jefferson T, del Mar C. The imperative to share clinical study reports: Recommendations from the Tamiflu experience. PLoS Med 2012;9(4):e1001201. doi:10.1371/journal.pmed.1001201. 22505850 PMC3323511

[76] Schroll JB, Penninga EI, Gøtzsche PC. Assessment of adverse events in protocols, clinical study reports, and published papers of trials of orlistat: A document analysis. PLoS Med 2016;13(8):e1002101. doi:10.1371/journal.pmed.1002101. 27529343 PMC4987052

[77] Jefferson T, Jones M, Doshi P, Spencer EA, Onakpoya I, Heneghan C. Oseltamivir for influenza in adults and children: Systematic review of clinical study reports and summary of regulatory comments. Br Med J 2014;348:g2545. doi:10.1136/bmj.g2547. 24811411 PMC3981975

[78] Golder S, Loke YK, Wright K, Sterrantino C. Most systematic reviews of adverse effects did not include unpublished data. J Clin Epidemiol 2016;77:125–33. doi:10.1016/j.jclinepi.2016.05.003. 27259470

[79] Zorzela L, Golder S, Liu Y, Pilkington K, Hartling L, Joffe A, Quality of reporting in systematic reviews of adverse events: Systematic review. Br Med J 2014;348:f7668. doi:10.1136/bmj.f7668. 24401468 PMC3898583

[80] Golder S, Loke Y, McIntosh HM. Poor reporting and inadequate searches were apparent in systematic reviews of adverse effects. J Clin Epidemiol 2008;61(5):440–8. doi:10.1016/j.jclinepi.2007.06.005. 18394536

[81] Golder S, Loke YK, Wright K, Norman G. Reporting of adverse events in published and unpublished studies of health care interventions: A systematic review. PLoS Med 2016;13(9):e1002127. doi:10.1371/journal.pmed.1002127. 27649528 PMC5029817

[82] Golder S, Loke Y, McIntosh HM. Room for improvement? A survey of the methods used in systematic reviews of adverse effects. BMC Med Res Methodol 2006;6:2–7. doi:10.1186/1471-2288-6-3. 16441876 PMC1402311

[83] Cornelius V, Perrio M, Shakir SA, Smith L. Systematic reviews of adverse effects of drug interventions: a survey of their conduct and reporting quality. Pharmacoepidemiol Drug Saf 2009;18(September):1223–31. doi:10.1002/pds.1844. 19757414

[84] Li L, Xu C, Deng K, Zhou X, Liu Z, Busse JW, The reporting of safety among drug systematic reviews was poor before the implementation of the PRISMA harms checklist. J Clin Epidemiol 2019;105:125–35. doi:10.1016/j.jclinepi.2018.09.014. 30278212

[85] Hopewell S, Wolfenden L, Clarke M. Reporting of adverse events in systematic reviews can be improved: Survey results. J Clin Epidemiol 2008;61(6):597–602. doi:10.1016/j.jclinepi.2007.10.005. 18411039

[86] Etminan M, Carleton B, Rochon PA. Quantifying adverse drug events: Are systematic reviews the answer? Drug Saf 2004;27(11):757–61. 15350149 10.2165/00002018-200427110-00001

[87] Committee on Standards for Systematic Reviews of Comparative Effectiveness Research; Institute of Medicine of the National Academies Finding What Works in Healthcare: Standards for Systematic Reviews. Eden J, Levit L, Berg A, Morton S, editors. The National Academies Press; 2011. doi:10.1016/b0-32-300162-9/50007-6. 24983062

[88] Higgins J, Green S, eds. Cochrane Handbook for Systematic Reviews of Interventions. 5.1.0.; 2011. www.handbook.cochrane.org

[89] Center for Drug Evaluation and Research. Meta-Analyses of Randomized Controlled Clinical Trials to Evaluate the Safety of Human Drugs or Biological Products.; 2018.

[90] Cochrane Collaboration Cochrane Handbook for Systematic Reviews of Interventions. Higgins J, Green S, editors. Wiley; 2019. doi:10.1002/9780470712184.ch5.

