# Supplementary material for: Summaries of harms in systematic reviews are unreliable Paper 1: An introduction to research on harms
Source: J Clin Epidemiol. Author manuscript; Available in PMC 2023 Mar 1. (PMC9126149; doi:10.1016/j.jclinepi.2021.10.023)
Supplement: Appendix [file NIHMS1773790-supplement-Appendix.pdf]

APPENDIX – Additional details on harms

Relatedness of harms to interventions

While the definition of an adverse event does not require that the event be related to the intervention, attribution of relationship might be useful for determining causality when assessing the types and relative frequencies of specific harms that should expected with interventions.[1–4] Additionally, there are regulatory requirements for reporting harms when causal relationships are suspected with an intervention.[1]

Determining relatedness is subjective and there is little consensus and guidance on how it should be done.[5] Relatedness describes how "likely" the treating physician (or outcome adjudication committee, investigator, study personnel, etc.) believes that a harm was caused by the intervention.[2–4,6] In general, relatedness should be considered based on principles of causality including the temporal relationship to the intervention, other plausible explanation for the harm, and whether the harm was expected given the clinical course, comorbidities, and concomitant medications.[1–5] For newly developed drugs, preclinical research and prior experiences with similar drugs (e.g., in the same class) can inform expectations and the designation of relatedness. Different scales may be used in the classification, but generally, they follow an ordinal relationship from "Unrelated" to "Definitely related" (**Table**).[2,6] Interested readers should explore the various approaches to defining and classifying relatedness to better understand the challenges and nuance therein.

Table. Classification of relatedness of a harm to an intervention according to CTCAE

Relationship	Attribution	Description
Unrelated to intervention	Unrelated	The harm is *clearly **NOT** related* to the intervention
	Unlikely	The harm is ***doubtfully related*** to the intervention
Related to intervention	Possible	The harm ***may be related*** to the intervention
	Probable	The harm ***is likely related*** to the intervention
	Definite	The harm ***is clearly related*** to the intervention

Other dimensions of harms data

Whereas harms are often summarized in studies using counts of occurrence, or by estimating the effect for an intervention group compared with another group (e.g., risk difference, risk ratio),[7–10] ample research suggests that patients want more information than just the likelihood of selected harms.[11–20] For example, patients want information about the timing, recurrence, and duration of harms.[7-22] For a given intervention, some harms appear quickly, after very little exposure. Others take a long time to develop, following a certain dose or duration of exposure. Some harms occur once and then resolve completely, whereas others occur multiple times.

With multiple dimensions and many different types of harms according to affected body systems and severity, the overall magnitude of harm caused by an intervention is difficult to quantify and present. Quantification may be possible through sophisticated approaches involving aggregating harms into compound outcomes and converting experienced harms into utility measures–a task being explored somewhat by the Global Burden of Diseases project.[23] But these methods are not standardized and current approaches to summarizing and communicating harm may be better served by qualitative and descriptive presentation as opposed to quantitative measures.

Data visualization provides a richer method for presenting an overall summary of harms through multiple dimensions of harms data, as compared with tables which are typically included in clinical trial reports.[24–27] Approaches to visualizing harms include the Dot Plot,[21] Stacked Bar Chart,[28] Volcano Plot,[29] Heatmap,[30] Treemap,[31] and Tendril Plot.[32] Examples of these visualizations, as well as code to reproduce them in R, can be found in a public GitHub repository (github.com/rquresh/HarmsVisualization). Efforts are currently underway to assess the value of visualization approaches for communicating harms from the perspectives of different stakeholder groups.[8,10,33,34]

Collection of harms

Systematic assessment of harms

Benefit outcomes are typically pre-specified in studies. Usually, they are systematically assessed for all participants using the same scales and instruments at planned times. Pre-specification of harms is much more difficult because some harms are unexpected or unknown. When harms can be anticipated (e.g., using preclinical studies or studies of similar interventions), investigators might collect those harms systematically.[35,36] Systematically assessed harms are those that are planned to be recorded for all participants in a study using the same methods, like the methods used to assess potential benefits.[37,38] Like potential benefits, systematically assessed harms can be fully specified using five components of an outcome: (1) domain, (2) measure, (3) method of aggregation, (4) metric, and (5) timepoint **(Box)**.[35,37,39,40] Systematically assessed harms are typically assessed using explicit methods to ascertain the presence of the harm, such as questionnaires or laboratory tests at pre-planned intervals.[35,37,41] For systematically assessed harms that are dichotomous, studies observing no events might report that 0 events occurred. Additionally, systematic collection for harms may include richer data for analysis of outcomes that are not assessed as events. For example, the effect of a drug that might cause weight gain could be assessed continuously as the between-group difference in body mass index (BMI).

Box. Example complete definition for systematically assessed harm

Domain: Nervous system effects

Measure (Name of scale): Vertigo Symptom Scale – Short Form (Likert scale of experience: 0 (never), 1 (a few times), 2 (several times), 3 (quite often [every week]), and 4 (very often [most days]))

Measure (Subscale): “Dizziness”

Metric: Value at the end of the study

Method of aggregation: Categorical – Risk difference (Proportion of participants with Score ≥ 1)

Timepoint: 4 weeks

4.2 Non-systematic assessment of harms

Primary studies address the need to gather information about unanticipated events by assessing harms non-systematically. Unfortunately, non-systematic assessment also leads to major challenges in assessing harms. Non-systematic assessment relies on the spontaneous reporting of harms,[37,38] or collecting harms in response to open-ended questions like "Have you noticed any symptoms since your last visit?". The latter differs from the spontaneous reporting in which data on harms are collected passively if volunteered. Harms assessed non-systematically are more susceptible to information bias compared with efficacy outcomes and systematically assessed harms. Moreover, non-systematic assessment reduces comparability of results because of differences in ascertainment across studies.[35–38,41–43] There may also be a reduction in overall power for harms if harms go unreported in both groups because participants are not asked and events are therefore not detected. In contrast to systematically assessed harms, it might not be appropriate to assume that 0 events occurred in studies with no observed non-systematic harms. Lastly, information about non-systematically assessed harms reported in journal articles might not be as rich or statistically useful as information for systematically assessed harms. Previous work from the Multiple Data Sources in Systematic Reviews (MUDS) Study has shown that hundreds of unique harms may be non-systematically assessed over the course of a trial, and many of these are never reported in subsequent trial publications.[41–43]

SUPPLEMENTARY REFERENCES

1. US department of Health & Human Services. *CFR Title 21, Section 312.32. IND Safety Reporting*.; 2010. https://www.accessdata.fda.gov/scripts/cdrh/cfdocs/cfcfr/CFRSearch.cfm?fr=312.32

2. National Cancer Institute. *Adverse Event Reporting Requirements for DCTD (CTEP and CIP) and DCP INDs and IDEs*.; 2013.

3. International Conference on Harmonisation of Technical Requirements for Registration of Pharmaceuticals for Human Use. *Clinical Safety Data Management: Definitions and Standards for Expedited Reporting E2a*.; 1994. http://www.ich.org/fileadmin/Public_Web_Site/ICH_Products/Guidelines/Efficacy/E2A/Step4/E2A_Guideline.pdf

4. Chan AW, Tetzlaff JM, Gøtzsche PC, Altman DG, Mann H, Berlin JA, Dickersin K, Hróbjartsson A, Schulz KF, Parulekar WR, Krleza-Jeric K, Laupacis A, Moher D. SPIRIT 2013 explanation and elaboration: guidance for protocols of clinical trials. *Br Med J (Clin Res Ed)*. 2013;346:e7586. doi:10.1136/bmj.e7586

5. Le-Rademacher J, Hillman SL, Meyers J, Loprinzi CL, Limburg PJ, Mandrekar SJ. Statistical controversies in clinical research: Value of adverse events relatedness to study treatment: Analyses of data from randomized double-blind placebo-controlled clinical trials. *Ann Oncol*. 2017;28(6):1183-1190. doi:10.1093/annonc/mdx043

6. National Cancer Institute. *Common Terminology Criteria for Adverse Events*.; 2020. doi:10.32388/erjxiq

7. Ma H, Ke C, Jiang Q, Snapinn S. Statistical considerations on the evaluation of imbalances of adverse events in randomized clinical trials. *Ther Innov Regul Sci*. 2015;49(6):957-965. doi:10.1177/2168479015587363

8. Cornelius V, Cro S, Phillips R. Advantages of visualisations to evaluate and communicate adverse event information in randomised controlled trials. *Trials*. 2020;21(1):1-12. doi:10.1186/s13063-020-04903-0

9. Phillips R, Sauzet O, Cornelius V. Statistical methods for the analysis of adverse event data in randomised controlled trials: a scoping review and taxonomy. *BMC Med Res Methodol*. 2020;20(1):1-13. doi:10.1186/s12874-020-01167-9

10. Phillips R, Hazell L, Sauzet O, Cornelius V. Analysis and reporting of adverse events in randomised controlled trials: a review. *BMJ Open*. 2019;9(2):e024537. doi:10.1136/bmjopen-2018-024537

11. Hakone A, Harrison L, Ottley A, Winters N, Gutheil C, Han PKJ, Chang R. PROACT: Iterative design of a patient-centered visualization for effective prostate cancer health risk communication. *IEEE Trans Vis Comput Graph*. 2017;23(1):601-610. doi:10.1109/TVCG.2016.2598588

12. Saket B, Endert A, Stasko J. Beyond usability and performance: A review of user experience-focused evaluations in visualization. *ACM Int Conf Proceeding Ser*. 2016;24-October:133-142. doi:10.1145/2993901.2993903

13. Laurent J, Mcdevitt H. *Interactive Data Visualization of Adverse Events Clinical Trial Data with the D3.Js Script Library*.; 2014.

14. Duke JD, Li X, Grannis SJ. Data visualization speeds review of potential adverse drug events in patients on multiple medications. *J Biomed Inform*. 2010;43(2):326-331. doi:10.1016/j.jbi.2009.12.001

15. Tarn DM, Wenger A, Good JS, Hoffing M, Scherger JE, Wenger NS. Do physicians communicate the adverse effects of medications that older patients want to hear? *Drugs Ther Perspect*. 2015;31(2):68-76. doi:10.1007/s40267-014-0176-7

16. Basch E. The missing patient voice in drug-safety reporting. *N Engl J Med*. 2010;362(10):865-869. doi:10.1056/NEJMp0911494

17. Berry D, Michas I, Bersellini E. Communicating information about medication side effects: Effects on satisfaction, perceived risk to health, and intention to comply. *Psychol Heal*. 2002;17(3):247-267. doi:10.1080/08870440290029520

18. Berry DC, Raynor DK, Knapp P, Bersellini E. Patients' understanding of risk associated with medication use: Impact of European Commission guidelines and other risk scales. *Drug Saf*. 2003;26(1):1-11. doi:10.2165/00002018-200326010-00001

19. Mcneal TM, Colbert CY, Cable C, Mirkes CR, Lubowinski JG, Myers JD. Patients ' attention to and understanding of adverse drug reaction warnings. *Patient Intell*. 2010;2:59-68. doi:10.2147/PI.S9569

20. Franklin L, Plaisant C, Shneiderman B. An information-centric framework for designing patient-centered medical decision aids and risk communication. *AMIA Annu Symp Proc*. 2013;(1):456-465.

21. Amit O, Heiberger R, Lane P. Graphical approaches to the analysis of safety data from clinical trials. *Pharm Stat*. 2008;7(February):20-35. doi:10.1002/pst.254

22. Yeh S-T. Clinical adverse events data analysis and visualization. *Pharm Ind SAS users Gr Conf Proc*. Published online 2007:PO10.

23. Murray CJL, Lopez AD, Mathers CD, Stein C. *The Global Burden of Disease 2000 Project: Aims, Methods and Data Sources*. Vol November.; 2001.

24. Gaissmaier W, Wegwarth O, Skopec D, Mu A, Broschinski S, Politi MC. Numbers can be worth a thousand pictures: Individual differences in understanding graphical and numerical representations of health-related information. *Heal Psychol*. 2012;31(3):286-296. doi:10.1037/a0024850

25. Feldman-stewart D, Kocovski N, Mcconnell BA, Brundage MD, Mackillop WJ. Perception of quantitative information for treatment decisions. *Med Decis Mak*. 2000;20:228-238. doi:10.1177/0272989X0002000208

26. Galesic M, Garcia-Retamero R. Using icon arrays to communicate medical risks: Overcoming low numeracy. *Heal Psychol*. 2009;28(2):210-216. doi:10.1037/a0014474

27. Hawley ST, Zikmund-fisher B, Ubel P, Jancovic A, Lucas T, Fagerlin A. The impact of the format of graphical presentation on health-related knowledge and treatment choices. *Patient Educ Couns*. 2008;73:448-455. doi:10.1016/j.pec.2008.07.023

28. Chuang-Stein C, Xia HA. The practice of pre-marketing safety assessment in drug development. *J*

Biopharm Stat. 2013;23(1):3-25. doi:10.1080/10543406.2013.736805

29. Zink RC, Wolfinger RD, Mann G. Summarizing the incidence of adverse events using volcano plots and time intervals. *Clin Trials*. 2013;10(3):398-406. doi:10.1177/1740774513485311

30. Izem R, Sanchez-Kam M, Ma H, Zink R, Zhao Y. Sources of safety data and statistical strategies for design and analysis: Postmarket surveillance. *Ther Innov Regul Sci*. 2018;52(2):159-169. doi:10.1177/2168479017741112

31. Chazard E, Puech P, Gregoire M, Beuscart R. Using treemaps to represent medical data. *Stud Health Technol Inform*. 2006;124:522-527.

32. Karpefors M, Weatherall J. The Tendril Plot-a novel visual summary of the incidence, significance and temporal aspects of adverse events in clinical trials. *J Am Med Informatics Assoc*. 2018;25(8):1069-1073. doi:10.1093/jamia/ocy016

33. Phillips R, Cornelius V. Understanding current practice, identifying barriers and exploring priorities for adverse event analysis in randomised controlled trials: An online, cross-sectional survey of statisticians from academia and industry. *Br Med J Open*. 2020;10(6). doi:10.1136/bmjopen-2020-036875

34. Qureshi R, Chen X, Goerg C, Mayo-Wilson E, Dickinson S, Golzarri-Arroyo L, Hong H, Phillips R, Cornelius V, McAdams-DeMarco M, Guallar E, Li T. Comparing the value of data visualization methods for communicating harms in clinical trials. *[submitted]*. Published online 2021.

35. Mayo-Wilson E, Fusco N, Li T, Hong H, Canner JK, Dickersin K. Multiple outcomes and analyses in clinical trials create challenges for interpretation and research synthesis. *J Clin Epidemiol*. 2017;86:39-50. doi:10.1016/j.jclinepi.2017.05.007

36. Mayo-Wilson E, Li T, Fusco N, Bertizzolo L, Canner JK, Cowley T, Doshi P, Ehmsen J, Gresham G, Guo N, Haythornthwaite JA, Heyward J, Hong H, Pham D, Payne JL, Rosman L, Stuart EA, Suarez-Cuervo C, Tolbert E, Twose C, Vedula S, Dickersin K. Cherry-picking by trialists and meta-analysts can drive conclusions about intervention efficacy. *J Clin Epidemiol*. 2017;91:95-110. doi:10.1016/j.jclinepi.2017.07.014

37. Zarin DA, Tse T, Williams RJ, Carr S. Trial reporting in ClinicalTrials.gov – The Final Rule. *N Engl J Med*. 2016;375(20):1998-2004. doi:10.1056/NJMsr1611785

38. US department of Health & Human Services. Clinical trials registration and results information submission: final rule. *Fed Regist*. 2016;81(183):64981-65157. doi:10.17226/10028

39. Zarin DA, Tse T, Williams RJ, Califf RM, Ide NC. The ClinicalTrials.gov results database – Update and key issues. *N Engl J Med*. 2011;364(9):852-860. doi:10.1056/NEJMsa1012065

40. Saldanha IJ, Dickersin K, Wang X, Li T. Outcomes in cochrane systematic reviews addressing four common eye conditions: An evaluation of completeness and comparability. *PLoS One*. 2014;9(10):e109400. doi:10.1371/journal.pone.0109400

41. Mayo-Wilson E, Fusco N, Li T, Hong H, Canner JK, Dickersin K. Harms are assessed inconsistently and reported inadequately Part 1: Systematic adverse events. *J Clin Epidemiol*. 2019;113:20-27. doi:10.1016/j.jclinepi.2019.04.022

42. Mayo-Wilson E, Fusco N, Hong H, Li T, Canner JK, Dickersin K. Opportunities for selective

reporting of harms in randomized clinical trials: Selection criteria for non-systematic adverse events. *Trials*. 2019;20(1):553. doi:10.1186/s13063-019-3581-3

43. Mayo-Wilson E, Fusco N, Li T, Hong H, Canner JK, Dickersin K. Harms are assessed inconsistently and reported inadequately Part 2: Non-systematic adverse events. *J Clin Epidemiol*. 2019;113:11-19. doi:10.1016/j.jclinepi.2019.04.020
